# Valve Calcification in Aortic Stenosis: Etiology and Diagnostic Imaging Techniques

**DOI:** 10.1155/2017/5178631

**Published:** 2017-07-24

**Authors:** María Manuela Izquierdo-Gómez, Iván Hernández-Betancor, Javier García-Niebla, Belén Marí-López, Ignacio Laynez-Cerdeña, Juan Lacalzada-Almeida

**Affiliations:** ^1^Department of Cardiology, Hospital Universitario de Canarias, Tenerife, Spain; ^2^Servicios Sanitarios del Área de Salud de El Hierro, Valle del Golfo Health Center, El Hierro, Spain

## Abstract

Aortic stenosis is the most common valvulopathy in the Western world. Its prevalence has increased significantly in recent years due to population aging; hence, up to 8% of westerners above the age of 84 now have severe aortic stenosis (Lindroos et al., 1993). This causes increased morbidity and mortality and therein lies the importance of adequate diagnosis and stratification of the degree of severity which allows planning the best therapeutic option in each case. Long understood as a passive age-related degenerative process, it is now considered a rather more complex entity involving mechanisms and factors similar to those of atherosclerosis (Stewart et al., 1997). In this review, we summarize the pathophysiological mechanisms underlying the onset and progression of the disease and analyze the current role of cardiac imaging techniques for diagnosis.

## 1. Introduction

Although aortic valvular sclerosis and aortic stenosis (AS) have long been thought of as two independent entities, they are now considered to be different stages of the same process. This disease manifests initially as valve thickening caused by lipocalcified deposits, leading to progressive reduction of the valve orifice which, over time, causes hemodynamically significant stenosis.

Aortic valve sclerosis is present in approximately 20–30% of individuals aged over 65 years and in 48% of patients over 85 years, while significant stenosis affects 2-3% of those over 65 years of age and up to 8% of those over 85 years [1, 2]. Thus its incidence increases exponentially with age and hence was long considered a simple passive age-related degenerative process with calcium buildup. However, several studies have shown that, in addition to age, calcific aortic valve disease (CAVD) is related to the presence of cardiovascular risk factors such as male sex, arterial hypertension, diabetes mellitus, dyslipidemia, and smoking, sharing many similarities with the process that regulates atherosclerosis [1–8]. There is therefore a direct relationship between the presence of valvular calcium deposits and the development of coronary disease and cardiovascular events [8–11], to the point that some authors even consider aortic calcification a possible marker of atherosclerosis and subclinical coronary artery disease [10, 12]. In 1986, Roberts [3] suggested that the presence of aortic mitral and valvular annular calcification was a form of atherosclerosis and numerous authors have since demonstrated this fact [6, 13–16]. In the Cardiovascular Health Study, the presence of aortic sclerosis in patients without previous coronary disease increased the risk of myocardial infarction and cardiovascular mortality 1.4 and 1.5 times, respectively [10]. In another prospective study involving 1,980 patients with a mean age of 81 ± 8 years, the probability of suffering a new coronary event increased by 80% for those with aortic sclerosis [6]. In addition, patients presenting altered mineral metabolism with hypercalcemia or increased bone demineralization, such as osteoporosis, have a higher prevalence of CAVD and a greater degree of disease progression [17, 18]. Advanced chronic renal failure has also been associated with CAVD, although this association has not been demonstrated in the earliest stages of the disease [19].

Several studies have identified valvular calcification as a manifestation of generalized atherosclerosis [9, 16, 20–25], and cardiac imaging techniques have acquired an added prognostic role regarding ischemic events.

In view of these considerations and the fact that aortic valve calcification and atherosclerosis present common pathophysiological mechanisms, the appearance of CAVD can no longer be considered simply an age-related degenerative valvular process but rather an active highly complex process, of probable systemic etiology, which involves biochemical, immunological, and genetic factors in an interactive way [26].

## 2. Pathogenesis of CAVD

In the initial stage of the disease, there is a focal thickening of the valves with formation of calcium nodules that begins on the aortic valve side at the subendothelial level and gradually extends to the outer or fibrous layer. These valves remain flexible for a long time, so that their opening mechanism is not affected. With the passage of time, the areas of thickening converge in large calcified masses that end up protruding into the exit tract of the aortic valve, conferring greater stiffness to the valves and significantly decreasing the valvular area, thus interfering with its normal functioning. Sclerosis and valvular stenosis affect both patients with tricuspid aortic valves and those with bicuspid valves, the most frequent congenital anomaly of aortic valve. The prevalence of bicuspid aortic valves is difficult to determine, but it is estimated to affect 1 to 2% of the general population. Up to 70% of patients with bicuspid aortic valves have valvular stenosis and will require aortic valve replacement 1 to 2 decades earlier than those with a trivalve aortic valve. In these patients, it is thought that traumatic degeneration of the cusps occurs culminating with fibrous degeneration and subsequent calcification of the valve [26].

From the microscopic point of view, there are many similarities with the lesions observed in the earliest stages of atherosclerosis [6, 13–16]. These lesions, initially interspersed with areas of normal tissue, will eventually coalesce and are characterized by disruption of the basement membrane, with areas of inflammation and cellular infiltration, deposit of atherogenic lipoproteins, and participation of the active mediators of calcification [27, 28].

Nowadays CAVD is considered a complex process involving factors of initiation, endothelial dysfunction, inflammatory response, and oxidative stress that lead to remodeling and valvular calcification. The study of its pathogenesis should therefore be based on the identification of genetic, anatomical, and clinical factors predisposing to the onset and progression of the disease [26, 29–31], since knowledge of this aspect would facilitate the prevention and treatment of patients with CAVD, especially those at the earliest stage of clinical expression.

### 2.1. Mechanical Stress

Aortic valve is subject to large mechanical stresses throughout the cardiac cycle. In response to this mechanical loading, valves undergo constant renewal and this fact favors valve pathology. The highest stress occurs in the areas of flexion of the leaflet. In these areas of increased mechanical stress, the process begins and bending force, pressure, and shear forces will induce calcification by damaging the structural integrity of the leaflet tissue. At present it is considered that mechanical stimuli play an important role in valvular calcification.

### 2.2. Endothelial Dysfunction

This mechanical stress on the flexion zone of the valve causes erosions at the endothelium level leading to endothelial dysfunction. Valvular endothelial cells were long considered a layer of cells that performed only coating functions. Today, that layer is considered a barrier that protects against metabolic, mechanical, and inflammatory insults, and the loss of its functions is a key element in the development of atherosclerosis [32]. Endothelial damage favors increased cell permeability, adhesion, and proliferation, which facilitates the diffusion of lipids to the interstitial valvular tissue and subsequent deposition in areas of inflammation and calcification.

### 2.3. Lipoprotein Deposit and Oxidative Stress

Lipid deposit plays an important initiator role in the cascade of cellular signaling leading to valvular calcification. The lipoproteins involved in the process include low-density lipoproteins (LDLs) and lipoprotein A. These are atherosclerosis molecules that undergo oxidation with the release of free radicals which are highly cytotoxic and also capable of stimulating inflammatory activity and mineralization [26, 30, 33–35]. The increase in oxidative stress during this process is demonstrated by the reduction of normal levels of nitric oxide at the endothelium level [29, 33] and by the marked increase in free radicals such as superoxide and oxygen peroxide [36], which is explained by an alteration in the normal function of nitric oxide synthetase.

LDLs are phagocytized by macrophages into foam cells, the fundamental substrate of the atherosclerotic plaque [37]. With progressive lipid uptake, these macrophages begin an irreversible transformation process that ends with apoptosis. Apoptosis also causes the release of factors that promote atherogenesis and progression to the complicated plaque stage, characterized by the presence of necrotic areas. At this point, it should be mentioned that some histological differences have been found in CAVD lesions with respect to atherosclerotic lesions. This may be relevant from the pathophysiological point of view. Unlike atherosclerotic plaque where the nucleus is composed of lipids associated with foam cells and areas of necrosis, in calcified valves the lipids are deposited mainly in the subendothelial zone and to a lesser extent in the deeper areas. Lipid-laden macrophages are evenly distributed in areas where there are high lipid concentrations and no areas of necrosis [38, 39]. In atherosclerotic plaques, the toxic accumulation of oxidized LDL causes cell death leading to plaque fracture. This is the major event that precipitates the appearance of clinically relevant symptoms. However, this mechanism has not been demonstrated in the case of CAVD [39], where the onset of symptoms is conditioned by the progression of calcification and increased valve rigidity.

Due to their proinflammatory and cell growth stimulating properties, oxidized LDLs are also capable of stimulating the formation of calcium nodules by activation of valvular fibroblasts. It has been proposed that accumulation of extracellular lipids and vesicles released by these activated fibroblasts constitute the nucleus for calcium deposition and subsequent formation of nodules [38].

Based on the association between CVAD and atherosclerosis and on the role of lipid deposition in this whole process, it has been hypothesized that hydroxymethylglutaryl coenzyme A inhibitor drugs may be useful to slow the progression of CVAD. Although some retrospective clinical studies have linked statin use with a slower progression of valvular stenosis, no prospective studies, except for a nonrandomized study on the use of rosuvastatin [40], have been able to demonstrate a beneficial effect at this level [41, 42].

### 2.4. Inflammation

Microscopically, the predominant inflammatory cells in CAVD are T lymphocytes and macrophages [43, 44]. These cells infiltrate and are deposited in the subendothelium contributing to the increase of proinflammatory cytokines and other enzymes that degrade the extracellular matrix [30]. They are also capable of inducing the transformation of fibroblasts into myofibroblasts with osteoblastic phenotype, which favor the formation of bone and calcium nodules [45]. The marked increase in cytokines such as interleukin-1 (IL-1) or tumor necrosis factor alpha (TNF-*α*) observed in calcified AS could demonstrate this fact [46]. In addition, it should be pointed out that there is pathological angiogenesis in CAVD, favored by mediators of inflammation, since these cause an increase in growth factors and endothelial transformation capable of inducing fibrosis and progression of calcification [47].

### 2.5. Alteration of the Extracellular Matrix and Calcification

In the more advanced phases of the disease there is remodeling of the extracellular matrix and calcification. Alteration of the matrix is promoted by the release of inflammatory cytokines that produce an increase in cellular proliferation expressed as increased matrix synthesis and activation of the extracellular matrix metalloproteinases, which not only favor the degradation of all its components but also directly promote the proliferation of fibroblasts, leading to an increase in fibrosis [48]. Increased fibrosis, along with the accumulation of calcium, contributes to valvular thickening and rigidity which lead to valvular stenosis.

Aortic calcification is a very complex active process involving the production of proteins that promote tissue calcification. In fact, extracellular matrix proteins normally found in bone, such as osteocalcin, osteopontin, and osteonectin, can also be found in calcified valves [49, 50]. This presence reveals pathological calcification and bone formation at the valve level. In short, this process involves different mechanisms of bone mineralization and resorption that begin at the level of the fibrous layer where some fibroblasts differentiate into myofibroblasts [30]. As a consequence of this differentiation, there is increased expression of proteins related to bone formation and the regulation of calcification at the level of the extracellular matrix. Although many proteins are implicated, the following are considered to play a key role: osteopontin, bone-forming proteins 2 and 4, osteoprotegerin, ligand-activating factor, and NF-*κ*B ligands (RANK and RANKL) [39]. All this results in the osteoblastic transformation of myofibroblasts and the cells of the valvular interstitium, which gives rise to nodules of calcification [45]. This differentiation involves multiple signaling pathways that could become potential therapeutic targets to attempt to control the progression of the disease [51, 52].

In addition, some authors suggest that valvular calcification is not only explained by an inflammatory process; they propose the intervention of so-called autoreplicative calcifying nanoparticles that have been detected in the calcified valves of patients with AS [53, 54].

### 2.6. Activation of the Renin-Angiotensin-Aldosterone System

In CAVD there are changes in the renin-angiotensin-aldosterone system that contribute to the pathogenesis of the lesion. These alterations affect the angiotensin-converting enzyme (ACE) and angiotensin II and angiotensin I receptors; they are related to increased LDL uptake, inflammation, and profibrotic state [55].

Treatment with drugs that block the renin-angiotensin-aldosterone cascade was long thought to have a beneficial effect in this group of patients, and, in fact, a retrospective study did show slower progression of calcification [56]. Unfortunately, the use of this group of drugs has not yet been shown to favorably modify the prognosis of these patients or help halt the hemodynamic progression of the disease [57].

### 2.7. Genetic Factors

Currently, there is evidence to show that genetic factors are involved in the development of CAVD. Several genetic polymorphisms have been identified in patients with the disease, such as the mutation of the gene encoding for the vitamin D receptor [58] or the gene coding for the synthesis of apolipoproteins responsible for the individual's lipid load [59]. Another polymorphism that has been extensively studied is that of the transcriptional factor NOTCH 1 which regulates the process of osteogenic differentiation. Under normal conditions, this pathway is responsible for inhibiting the differentiation of osteoblasts, so that mutations at this level promote such differentiation, favoring calcification and the appearance of CAVD.

In summary, the study and the detection of these polymorphisms should allow us to identify patients at risk of developing CAVD and apply early preventive treatment.

## 3. Imaging Techniques for the Study of CAVD

Cardiac imaging techniques play a key role in the study of CAVD. In addition to confirming the diagnosis and estimating the degree of severity, they have great prognostic utility. This allows evaluation of the possible functional repercussion and follow-up of patients at risk in order to plan the optimum moment for valve replacement.

Among these imaging techniques, echocardiography remains the cornerstone. However, in recent years others, such as cardiac magnetic resonance imaging or computed tomography, have also demonstrated their usefulness, obviating the technical limitations of echocardiography and providing additional information on some anatomical and functional aspects which are especially relevant when considering transcatheter aortic valve implantation (TAVI) or surgical repair techniques [60].

### 3.1. Echocardiography

**Figure 1:**
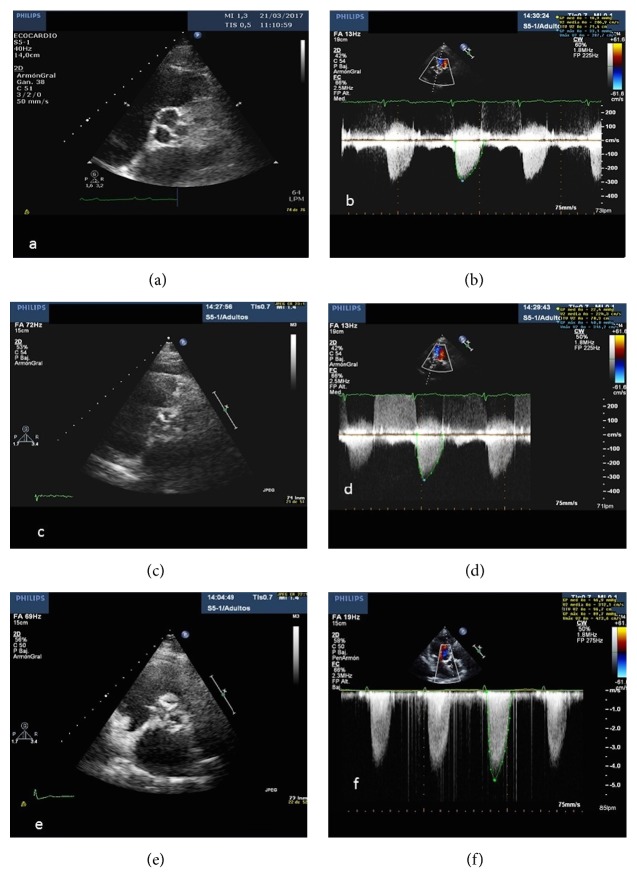
Two-dimensional echocardiogram, short axis view, and continuous Doppler showing mild (a and b), moderate (c and d), and severe (e and f) aortic valve stenosis, respectively.

Transthoracic echocardiography (TTE) is the method of choice in the study of CAVD. It is a noninvasive, safe, and widely available technique that allows very early diagnosis of valvular alterations that occur as a consequence of calcium deposition, characterized by valvular thickening or sclerosis. Initially this does not cause significant hemodynamic alteration, but as the disease progresses there is increasingly significant valve stenosis with great functional repercussion which usually coincides with the appearance of symptoms (Figure 1).

TTE not only allows us to evaluate valve morphology, the etiology of the stenosis, and the degree of severity but also provides additional information about other important parameters such as ventricular function, cavity dimension, or pressure at the level of the pulmonary artery which may be altered in response to pressure overload and as a consequence of a complex process of ventricular remodeling [35]. Taking into account all of the above, current clinical practice guidelines include the need to plan periodic echocardiographic follow-up of these patients, and this constitutes an important factor in decision-making regarding aortic valve replacement [61].

**Figure 2:**
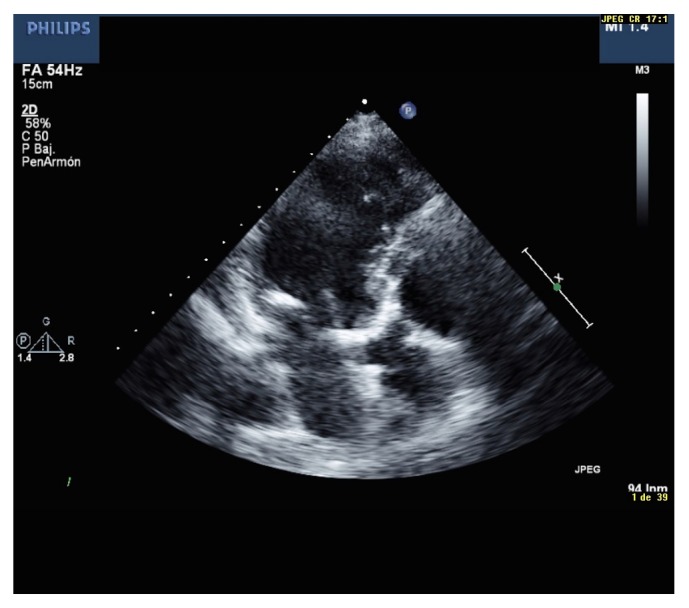
Transthoracic echocardiography, three-chamber view: intense calcification of aortic and mitral valves.

As has been demonstrated in previous studies, the degree of valvular calcification is a relevant predictor of heart failure, need for valvular replacement, and death [62]. From the morphological point of view, the extent and severity of valvular calcification can be graded semiquantitatively as mild (presence of small hyperechogenic areas with little acoustic shadowing), moderate (multiple large areas with dense echogenicity), or severe (extensive thickening with increased echogenicity and high acoustic shadowing) [63] (Figure 2).

However, in the current study of CAVD, in addition to estimating the extent and distribution of calcium, it is essential to make an accurate hemodynamic estimation and to classify the severity of valvulopathy. For this, there are a series of recommendations and standard measures that should be included in all echocardiographic studies, such as peak velocity, mean transvalvular pressure gradient, and left ventricular outflow tract diameter (LVOT) [61]. These measures allow the aortic valve area (AVA) to be calculated by applying the continuity equation, based on the principle that the ejection volume at the level of the LVOT should be equal to the stroke volume passing through the stenotic valve. Accordingly, aortic sclerosis is considered to exist when peak aortic jet velocity is ≤2.5 m/s. Severe AS is considered to exist when the peak velocity ≥ 4 m/s, the mean gradient ≥ 40 mmHg, and AVA is <1 cm^2^.

Calculating AVA by echocardiography has certain limitations. In addition to the difficulty of accurately measuring LVOT in some cases, a well-known source of error, velocity and pressure gradients are flow-dependent parameters, so that certain hemodynamic situations could alter its value. An example is patients with low flow at the valve level (stroke volume index <35 mL/m2), who may present severe AS with AVA <1 cm2, but with a peak velocity and a mean gradient of <4 m/s and <40 mmHg, respectively. In this case, there is a discrepancy between the parameters that define the severity, which could hinder decision-making, and this circumstance obliges us to integrate the echocardiographic findings with all the available clinical information before establishing the indication for surgery. In recent years, new concepts and special considerations have emerged in the assessment of severe AS, which involve knowing the state of ventricular function and flow. Integrating all this information facilitates understanding these discordant findings (once other sources of error have been ruled out) observed in certain subgroups [60, 63] and which we briefly describe below.

**Figure 3:**
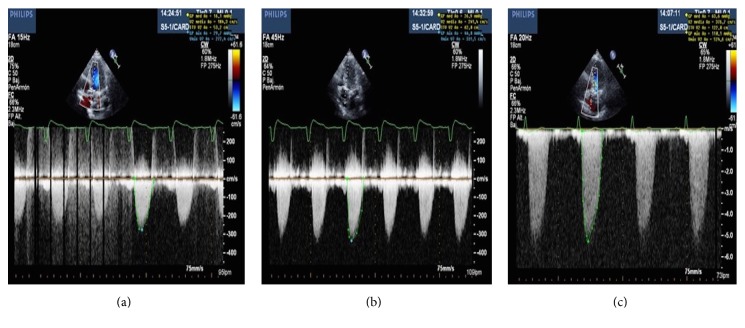
Patient with true low gradient severe aortic stenosis showing increased aortic valve gradient with continuous Doppler during dobutamine infusion at 10 g/kg/min (a), 15 g/kg/min (b), and 20 g/Kg/min (c). Aortic valve area remained below 0.5 cm^2^ during infusion.

*(i) Severe AS with Low Flow, Low Gradient, and Reduced Left Ventricular Ejection Fraction (LVEF).* Patients with severe AS and left ventricular systolic dysfunction have a low beating volume conditioned by altered contractility. As a consequence, despite having AVA < 1 cm^2^, the mean gradient is low (<40 mmHg) [64, 65]. It is important to differentiate between this circumstance and “pseudosevere” AS, where the existence of important ventricular dysfunction prevents the generation of sufficient and necessary energy to allow valve opening, in which case we obtain an AVA < 1 cm^2^ with a mean gradient of <40 mmHg; the consequence is a falsely small valvular area. After valve replacement surgery, patients with “true” severe AS, ventricular dysfunction and low gradient show improved LVEF, whereas patients with “pseudosevere” AS do not regain ventricular function, thus not benefiting from surgery [66]. In order to distinguish between the two situations, a stress echocardiogram with low-dose dobutamine [61, 67] is recommended; an increase >40 mmHg (or >4 m/s peak velocity) during infusion, without an increase in AVA, suggests the presence of “true” severe AS (Figure 3).

The use of this protocol also allows us to estimate the LV contractile flow reserve. When this parameter is reduced (increased ejection volume less than 20% with dobutamine infusion), it serves as a predictor of surgical mortality [68, 69].

*(ii) Severe AS with Low Flow, Low Gradient, and Preserved LVEF.* This picture is also known as paradoxical low flow, low gradient severe AS. It is characterized by the presence of severe AS with AVA <1 cm2, a mean gradient of <40 mmHg, and a peak velocity of <4 m/s despite a normal LVEF (≥50%) [70, 71]. Recently described, it usually occurs in patients with pronounced concentric left ventricular remodeling, which conditions a small ventricular cavity and a physiopathological restriction of the LV that causes filling problems and, consequently, a low ejection volume. In this scenario, stress echocardiograms have not proved useful. However, it seems that an analysis of valvular calcium using CT scan may aid in the diagnosis [72], since surgery would also be indicated in these patients [73].

*(iii) Severe AS with Normal Flow, Low Gradient, and Preserved LVEF.* Recent investigations have identified a group of severe AS patients with AVA <1 cm2 and mean gradient of <40 mmHg, despite having normal LVEF and flow. It is thought that such small valve area is probably due to measurement error, a low body surface area, or inconsistency in the cut-off points established by the guidelines [74] and that they are really patients with nonsevere AS. This is supported by studies that demonstrate that these patients have similar prognosis and outcome to those with moderate stenosis [75]. However, close follow-up with periodic reevaluation is recommended in these cases, especially if they are symptomatic.

**Figure 4:**
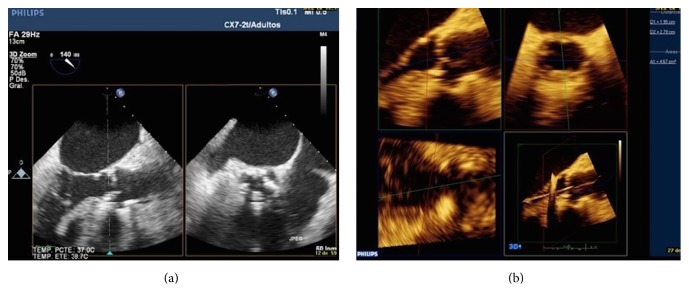
AVA calculation with 2D (a) and 3D transesophageal echocardiography (b).

Two- and three-dimensional transesophageal echocardiography (TEE) also plays a role in the study of CAVD. These techniques, though not usually performed routinely, are especially useful in the case of poor acoustic window and represent a good alternative to AVA determination by planimetry. In addition, TEE allows a more accurate measurement of aortic root size and valve ring dimensions, which are essential when planning surgery, especially before TAVI [76] (Figure 4).

**Figure 5:**
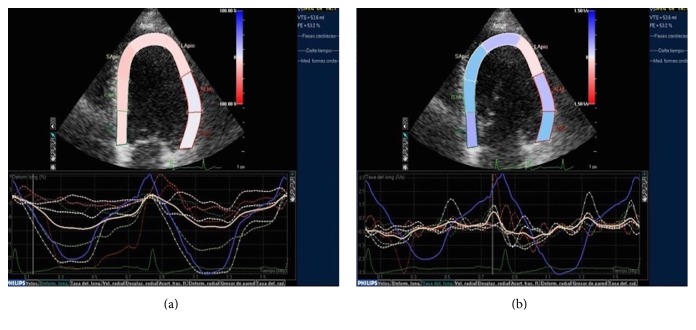
Strain (a) and strain rate (b) of the left ventricle with severe hypertrophy and normal deformity.

To conclude with the echocardiographic evaluation, one must mention the utility of speckle tracking in the study of aortic valvulopathy. This novel technique allows evaluation of myocardial function by analysis of the parameters of ventricular deformity (strain and strain rate). The architecture of the ventricular myocardium is complex and characterized by the presence of a bundle of fibers distributed in different layers and oriented longitudinally and circumferentially [77]. Ventricular systolic function depends on the contraction of the whole bundle of fibers. Classically, the parameter used to assess ventricular function is LVEF. However, LVEF can be preserved until the end stages of the disease. In contrast, strain is a more sensitive parameter in the identification of alterations in myocardial contractility. It allows a very early analysis of the changes in the regional and global myocardial function that occur as a consequence of the increase in afterload and, as it progresses, the degree of severity of the AS even before LVEF is affected. Thus, alteration in its value indicates incipient affectation of ventricular function and hence its usefulness [78]. This has prognostic implications since it has been shown that patients with altered global longitudinal strain and severe AS, despite normal LVEF and an absence of symptoms, have a worse prognosis than those with normal global longitudinal strain values [79, 80]. In addition, global longitudinal strain alteration could reflect the presence of extensive myocardial fibrosis, also associated with worse prognosis [81]. Nevertheless, these parameters have not yet been formally included in the current clinical practice guidelines, although some recent multimodal imaging guidelines in the study of AS already define their role and advocate their inclusion to obtain more complete information, before deciding on the therapy to be applied in each case [78, 81] (Figure 5).

### 3.2. Cardiac Magnetic Resonance Imaging (CMRI)

Cardioresonance is an emergent, noninvasive, and safe technique, without X-ray exposure; it is very precise and has high spatial resolution. Accuracy and reproducibility are high, even in patients with inadequate echocardiographic images, which currently make it the reference technique for the noninvasive study of LV dimensions and mass and global and regional left ventricular function [82, 83]. This includes patients with CAVD, so CMRI allows us to analyze the effects of ventricular remodeling that occur as the disease progresses and the increasing degree of severity of the stenosis [60]. It is thus a useful tool in the follow-up of these patients [84]. CMRI also provides information about valvular morphology, in some cases allowing us to estimate AVA by means of planimetry and adds information about the dimensions of the aorta when considering surgery.

In addition, CMRI allows a more functional analysis based on phase contrast pulse sequences. This type of sequence involves the acquisition of two types of images, one with velocity coding (phase sequences) and the other with purely anatomical images (magnitude sequences), acquired simultaneously. In these sequences, stationary tissue is displayed as gray while the flow through the region of interest in the positive direction is displayed as white and the flow in the negative direction appears black. Velocity can be coded in planes that are perpendicular to the axis of the flow (through the plane) or in parallel planes (in the plane). In this way, velocity and volume can be measured in any vessel at any point of the cycle and the maximum jet speed of the stenosis can be estimated [85]. Applying the modified Bernoulli equation and the continuity equation, the maximum gradient through the stenosis and the valvular area can be obtained, respectively, achieving good correlation with echocardiography and high reproducibility with CMRI [86, 87]. Without performing hemodynamic calculations, the detection of flow artifacts in film sequences also suggests the presence of stenosis.

**Figure 6:**
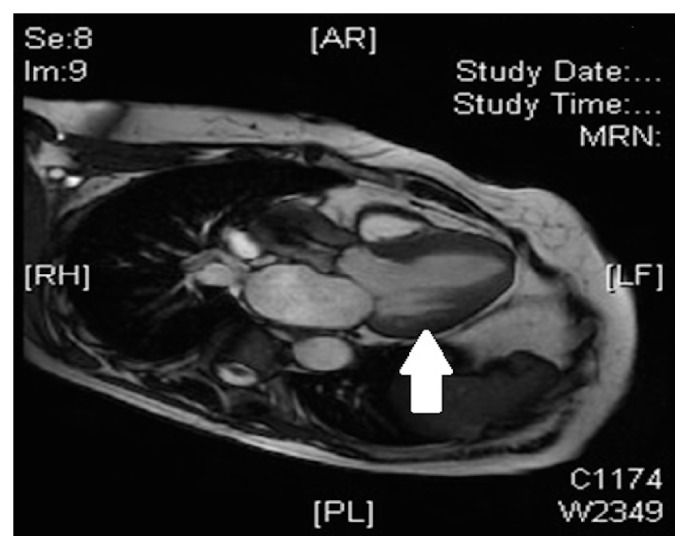
Gadolinium contrast cardiac magnetic resonance image showing left ventricular myocardial fibrosis in a patient with severe aortic stenosis and ventricular hypertrophy (arrow).

Apart from being an excellent technique for studying contractility, one of the main advantages of CMRI over other imaging techniques is its capacity for tissue characterization [83]. With gadolinium administration, late enhancement, and T1 mapping, we can differentiate the healthy myocardium from areas that present myocardial fibrosis. The presence of extensive myocardial fibrosis is a prognostic indicator of worsening functional class, ventricular dysfunction, and poor recovery rates after valve replacement surgery [88], and the degree of preoperative fibrosis constitutes an independent predictor of postoperative mortality [89]. The main drawbacks of CMRI are the lack of availability and the cost of the technique (Figure 6).

### 3.3. Computed Tomography (CT)

Multislice CT provides accurate anatomical images of the aorta root and valvular orifice. Increasing use of this technique before TAVI to measure LVOT and annular size has demonstrated that LVOT is generally not circular but eccentric, which means that AVA is commonly underestimated when measured by echocardiography [90]. Calculating the size of the LVOT by means of planimetry using CT would increase the accuracy of AVA calculation.

**Figure 7:**
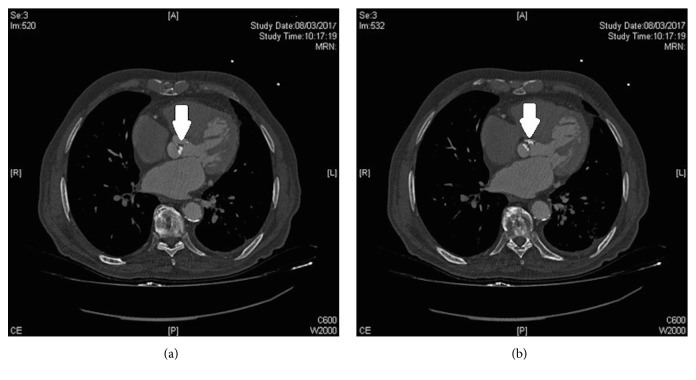
Chest computed tomography scan showing quantification of aortic valve calcification (a and b) (arrows).

This technique offers the advantage of quantifying the calcium load at the valve level. For this, the Agatston score is used, which shows good correlation with echocardiographic measurements and when high, is an important predictor of poor prognosis and disease progression [91]. A recent study [92] has suggested a cut-off point (≥2065 Agatston units for men and ≥1274 Agatston units for women) in order to distinguish severe from moderate AS. This could be very useful as an adjuvant measure in controversial patients, with, for example, severe AS, low flow, and low gradient, so this additional measure, which is also flow-independent, could help to define the degree of severity. However, further studies are needed to confirm its predictive value (Figure 7).

## 4. Conclusions

CAVD is highly prevalent. Long understood as a passive process, it is now known to be complex and one which involves pathophysiological mechanisms similar to those of atherosclerosis. Understanding these mechanisms could help to establish new therapeutic targets that might allow us to halt or at least slow down the progression of the disease.

For the diagnosis of CAVD we have different imaging tests, where echocardiography represents a fundamental tool. From the echocardiographic point of view, it is important to estimate the degree of severity, so AVA calculation obtained using flow-dependent parameters remains the main measure. However, certain hemodynamic situations, such as the presence of low flow or left ventricular dysfunction, have been shown to generate inconsistencies in the measurements, so nowadays it is considered essential to analyze such circumstances in each patient. In addition, new parameters have appeared in the evaluation of LV ventricular function, such as strain and strain rate, which could be useful to detect subclinical stages of ventricular dysfunction. Only in this way can a more complete evaluation be guaranteed, which is of great importance since, according to current clinical practice guidelines, the indication for aortic valve replacement will depend fundamentally on the combination of clinical status and echocardiographic findings.

Despite the importance of echocardiography, the limitations of this technique in many cases oblige us to complete the information with other imaging techniques such as CMRI and CT. The former allows evaluation of ventricular remodeling, using parameters such as maximum velocity and mean aortic valve gradient, and AVA calculation using phase contrast sequences. But one of its greatest advantages is that CMRI enables the detection of intramyocardial fibrosis. CT provides very accurate anatomical images of the valve orifice and allows quantification of valvular calcium load. Both techniques are of great help in the prior evaluation of candidates for TAVI. Given these considerations, the challenge now is to integrate all the information provided by each of these techniques to help clinicians in their diagnostic and therapeutic approach in a timely and appropriate manner.

## Abbreviations

AS: Aortic stenosis
AVA: Aortic valve area
CAVD: Calcific aortic valve disease
CMRI: Cardiac magnetic resonance imaging
CT: Computed tomography
LV: Left ventricle
LVEF: Left ventricular ejection fraction
LVOT: Left ventricular outflow tract diameter
LDL: Low-density lipoprotein
TAVI: Transcatheter aortic valve implantation
TTE: Transthoracic echocardiography
TEE: Transesophageal echocardiography.
